# The Clinical and Radiological Characteristics of Inflammatory Myofibroblastic Tumor Occurring at Unusual Sites

**DOI:** 10.1155/2018/5679634

**Published:** 2018-05-14

**Authors:** Xuewei Zeng, Huayi Huang, Jun Li, Jiayou Peng, Jiaxiong Zhang

**Affiliations:** Department of Radiology, Foshan Hospital of Traditional Chinese Medicine, Foshan 528000, China

## Abstract

Inflammatory myofibroblastic tumor (IMT) can occur rarely in the soft tissue or joint of the limb. We retrospectively collected IMT cases of these rare sites and analyzed their clinical and imaging appearance. Thirteen cases of IMT were clinically diagnosed and underwent surgical procedures, pathological analyses, and postsurgical follow-up in our two hospitals. Other than one case of IMT of the bladder wall that presented with gross hematuria, none presented with local swelling, fever, or weakness. All the cases of IMT occurring at the bone showed destruction and parosteal soft tissue masses. The boundaries between the mass and normal bone were vague, without calcifications or any periosteal reaction. Five cases of IMF showed continuous enhancement on CT; seven cases demonstrated iso- or hyposignal intensity on T1WI; one case showed hypersignal intensity on T1WI, and eight cases demonstrated a hypersignal intensity signal on T2WI. All the masses located in soft tissues showed clear and sharp boundaries with different sizes of the swelling regions surrounding muscle interspaces. Three cases showed homogeneous enhancement, one case demonstrated heterogeneous enhancement, and two cases showed edge enhancement on enhanced MRI scans. On pathology, all the lesions showed an absence of a pseudocapsule, and four cases of ALK were positive. The radiological manifestations of IMT located at the soft tissue and bones were similar to benign tumors in shape; however, peritumoral edema, parosteal soft tissue, and the invasive rim of IMT are similar to the features of malignant tumors. Different radiological methods should be used to obtain an accurate diagnosis.

## 1. Introduction

Inflammatory myofibroblastic tumor (IMT) is a type of tumor comprised of differentiated myofibroblastic spindle cells, infiltrated by many inflammatory cells, and is usually accompanied by conspicuous lymphoplasmacytic infiltrates and a myxoid stromal background. The spindle to epithelioid tumor cell exhibits a myofibroblastic immunophenotype with expression of smooth muscle actin, calponin, and/or desmin [1].

Currently, the status of IMT as either a reactive lesion or true neoplasm has not been defined. The Fourth Edition of the World Health Organization (WHO) Classification of Tumors of Soft Tissue and Bone (2013) [2] classified IMT as an intermediate neoplastic lesion (aggressive, with occasional metastases). It can occur anywhere in the body but is most frequently documented in the lung, retroperitoneum, and gastrointestinal tract [3], and it is rarely located in the soft tissue of the extremities, bones, or joints. A proportion of IMT cases exhibit local recurrences and occasional distant metastases which cannot be well explained by an inflammatory reaction [4].

The imaging manifestations of IMT are variable and display no characteristic representation on CT or MR imaging, despite recent improvements in imaging technology [5]. IMT have a similar appearance to other typical spinal epidural lesions, such as meningioma, lymphoma, and metastatic tumor [6]. In clinical practice, nonspecific manifestations present a great challenge to diagnosis and treatment of the condition.

In this article, we reviewed the imaging features of thirteen diagnosed cases of IMT situated in uncommon sites, to improve understanding of the disease and to propose a set of diagnostic criteria to distinguish between IMT and other mesenchymal neoplasms.

## 2. Methods and Materials

### 2.1. Patient Data

A total of thirteen IMT cases identified from April 2005 to July 2015 at two hospitals (including our own) were enrolled. All the cases were clinically diagnosed and underwent surgical procedures, pathological analysis, and postsurgical follow-up in the two hospitals.

### 2.2. Imaging Acquisition

X-ray or computer tomography (CT) exams were performed on seven cases before surgery. Dual energy CT (Siemens SOMATOM Definition) images were acquired with the following parameters: slice thickness of 5–10 mm; tube voltage of 120 kV; tube current of 559 mA; and pitch of 3.2; in addition, the gantry was perpendicular to the CT table. Multiplanar reformatting of the CT images was performed on a workstation (Advantage Workstation 4.3; GE Healthcare, Waukesha, WI, USA).

Magnetic resonance imaging (MRI) with and without contrast was performed on six cases on a 3.0T MRI scanner (GE Signa Excite). Dedicated coils were used for imaging different areas of the body and regions of interest. The MR images were acquired using spin echo sequences. The parameters for the axial view were as follows: T_1_WI: TR/TE 440/8.2 ms; T_2_WI: TR/TE 4000/142.5 ms; slice thickness: 5 mm; NEX: 4.0; FOV: 38 cm × 38 cm; and matrix: 256 × 224~512 × 446. For the fat-suppressed T1-weighted transverse images, gadopentetate dimeglumine (Magnevist, Schering, Berlin, Germany) was intravenously injected at a dose of 0.1 mmol/kg per patient and an injection rate of 2.0 ml/s. The parameters for the axial view were as follows: T_1_WI: TR/TE 560/8.0 ms; slice thickness: 6 mm; FOV: 38 cm × 38 cm; and matrix: 320 × 192~512 × 446. The parameters for the coronal view were as follows: T_1_WI: TR/TE 560/8.2 ms; slice thickness: 5 mm; FOV: 40 cm × 40 cm; and matrix: 320 × 192~512 × 446.

### 2.3. Image Analysis

All the images were analyzed by two board-certified radiologists specialized in musculoskeletal imaging. On CT imaging, the tumor location, morphology, size, margins, density, and the presence of calcification in the tumor were evaluated. On MR imaging, the tumor morphology, edge, signal intensity and enhancement, necrosis, hemorrhage, and peritumoral edema were evaluated. All the imaging findings were correlated with the pathological analysis.

### 2.4. Pathological Analysis

Tumor specimens obtained after surgical resection were fixed in 10% formaldehyde solution for 24 hours for dehydration, and the paraffin-embedded specimens were sliced and stained with hematoxylin and eosin (H&E). Immunohistochemistry streptavidin-biotin staining (S-P) link staining with 3,3′-diaminobenzidine (DAB) color rendering was performed on some specimens. The finding of a cytoplasmic brown precipitate was deemed positive. Histopathological characteristics were evaluated by a pathologist specializing in musculoskeletal medicine.

## 3. Results

### 3.1. Clinical Data

Among the thirteen cases of IMT studied, seven were male and six were female. The ages ranged from 3 to 59 years, with a mean age of 32.7 years. The medical history ranged from 20 days to 8 years, and 3 (23.1%) cases exhibited local tumor recurrences after surgical resection. In one case, tumors recurred eight times within eight years following the surgery. The maximum diameter of the mass was 2.0–~8.7 cm, and the mean maximum diameter was 4.8 cm. The locations of tumors in the thirteen cases are shown in Table 1. Other than one case located in the bladder wall that presented with gross hematuria, none of the cases presented with local swelling, fever, or weakness. The laboratory examination of six cases showed lower or marginally lower Mean Corpuscular Volume (MCV), Mean Corpuscular Hemoglobin (MCH), and Mean Corpuscular Hemoglobin Concentration (MCHC), and four showed a higher platelet count.

### 3.2. X-Ray and CT Features

As shown in Table 1, four cases were localized to the bones: two in the frontal bone, one in the maxilla, and one in the intertrochanteric femur. The lesions in the frontal bone showed destruction of all or most of the inner and outer plates, showed clear and sharp boundaries, and showed no calcification or ossification (Figures 2 and 6). The lesions in the femur demonstrated expansive growth, with internal ground glass opacities, an unbroken sclerotic rim, and no cortical involvement (Figure 5). One case localized in the bladder showed bladder wall involvement and pelvic cavity adhesions. Another two cases located on the body surface showed surrounding tissue adhesions (Figure 3(a)), and the remaining four cases demonstrated clear and sharp boundaries. No calcification was found in the other seven cases of IMT.

On unenhanced CT scanning, the mean CT numbers of the seven cases were in the range 15–67 HU. After contrast-enhanced scanning, the tumor enhancement of one case (Case 1) was not apparent as it measured less than 10 HU. The remaining five cases demonstrated mild-to-moderate enhancement, ranging from 10 to 60 HU. On the delayed contrast scans, the CT number continued to rise.

### 3.3. MRI Features

**Figure 1:**
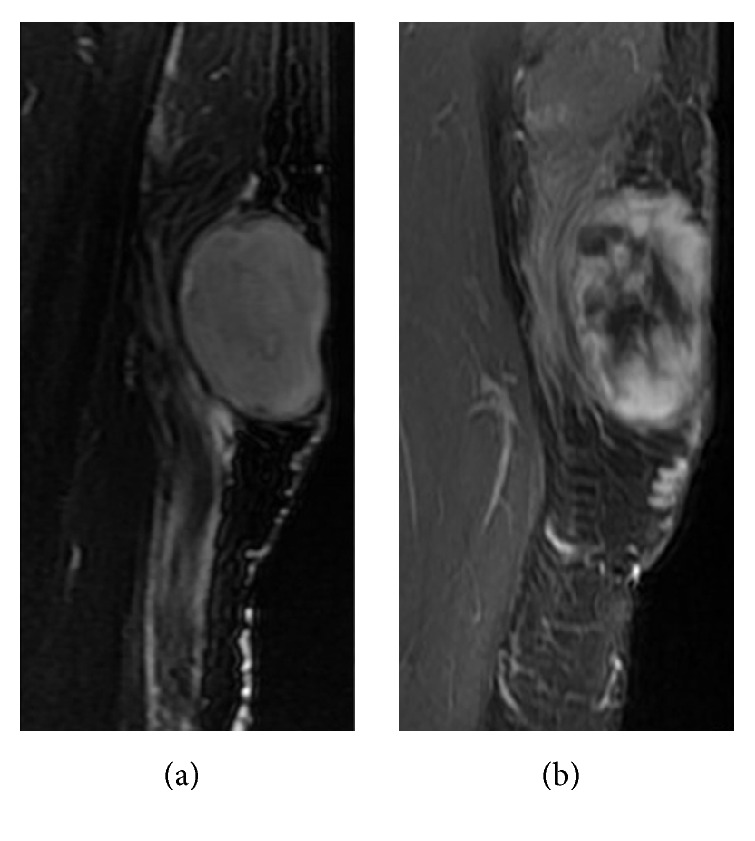
Images obtained from a 44-year-old female with IMT of the right thigh. (a) An oval-shaped soft tissue mass in the right thigh with long T2 was observed. (b) An enhanced MRI scan revealed heterogeneous enhancement.

**Figure 2:**
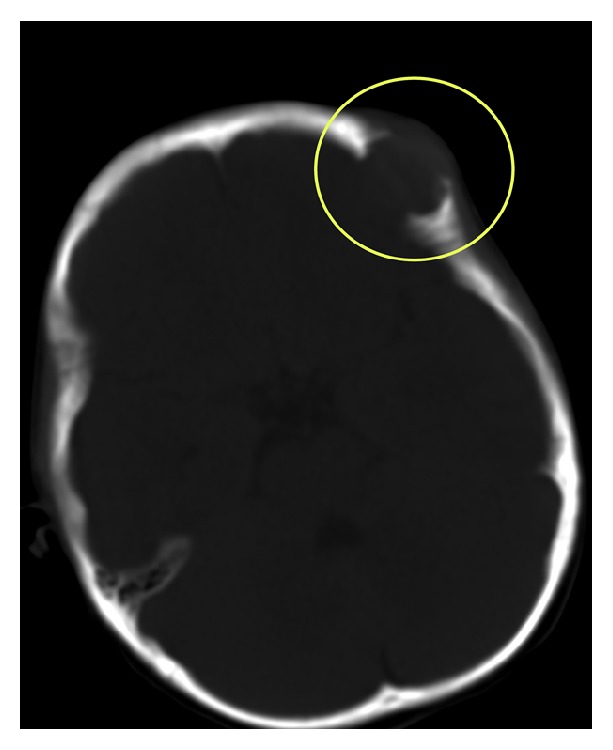
Images obtained from a 3-year-old female with IMT of the frontal bone. The CT scan showed destruction of the inner and outer plates, clear and sharp boundaries, and no calcification or ossification.

**Figure 3:**
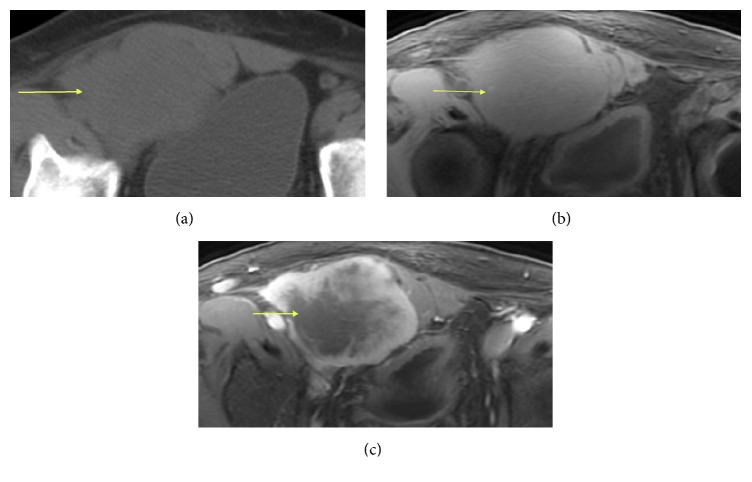
Typical radiological appearance of a 47-year-old man with IMT of the right groin. (a) An isodense, lobulated soft tissue mass in the right groin. (b) T2-weighted MRI showed a homogenous iso-signal mass. (c) Gadolinium-enhanced T1-weighted fat-suppression images showed heterogeneous enhancement (yellow arrow).

**Figure 4:**
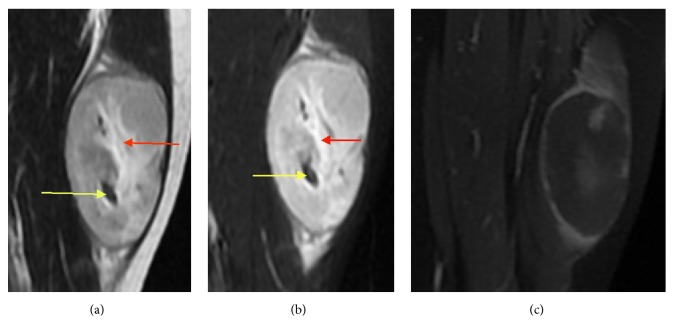
A 16-year-old female with IMT of the left thigh. (a) An oval-shaped soft tissue mass with short T1 and (b) long T2 signal was observed, with associated hemorrhage (red arrow) and vascular lesions (yellow arrow). (c) An enhanced MRI scan revealed rim enhancement.

**Figure 5:**
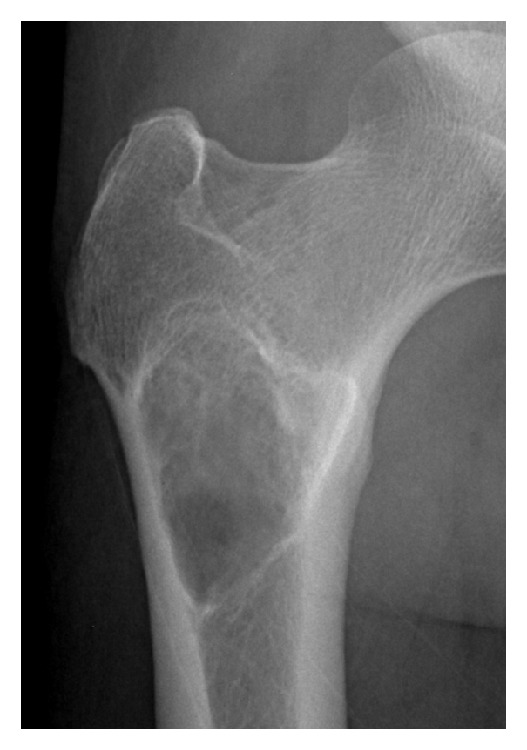
Images obtained from a 48-year-old male. Lesions in the femur demonstrated expansive growth, with internal ground glass opacities, an unbroken sclerotic rim, and no cortical involvement on X-ray.

**Figure 6:**
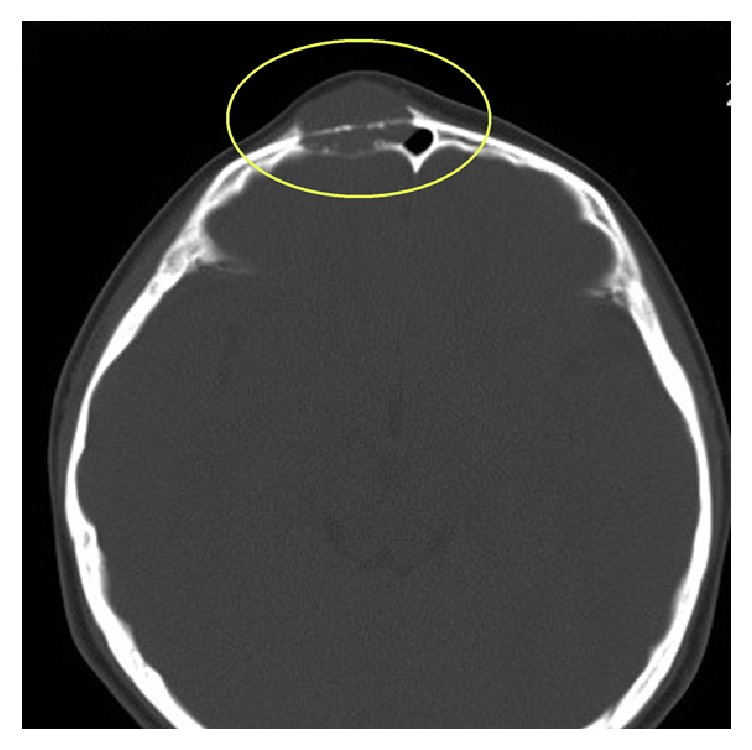
Images obtained from a 10-year-old female with IMT of the frontal bone. The CT scan showed destruction of the inner and outer plates, clear and sharp boundaries, and no calcification or ossification.

As shown in Table 1, eight cases received both noncontrast and contrast-enhanced MRI scans. On scanning, the masses of seven cases appeared homogeneously iso- or slightly hypointense, and the hemorrhagic lesion of one case appeared hyperintense on T1WI and T2WI (Figures 4(a) and 4(b)). The MR scans of the eight scanned cases all appeared hyperintense on T2WI (Figures 1(a) and 4(b)), without an obvious septate, necrotic, or cystic constituency. All the masses located in soft tissue showed clear and sharp boundaries, and different sizes of hyperintense swelling regions were seen in the surrounding muscle tissue and muscle interspaces. Enhanced CT scans demonstrated homogeneous enhancement in three cases, heterogeneous enhancement in one case (Figure 1(b)), and edge enhancement in two cases (Figures 3(c) and 4(c)).

### 3.4. Pathological Manifestations

Masses in all the cases appeared taupe and yellow-gray during the operation. The lesions showed the absence of a pseudocapsule and adhered to the surrounding tissues. Gross specimens showed the tumor to be jelly-like and soft on a cut surface, occasionally appearing with hemorrhagic lesions. Under a light microscope, the tumor cells in the mass were distributed unequally. The puff zone was rich in collagen fibers, which were low density and arranged in an irregular manner and also contained abundant vasculature. The tumor cells differed in shape and size. Multinuclear and polymorphic nuclei could be seen; however, mitotic figures were rare. There were also a few sporadic ganglion cells, each with a vacuole nucleus and an obvious nucleolus, involving surrounding fat and muscle tissue, along with the infiltration of neutrophils, lymphocytes, plasmacytes, and histiocytes. Seven patient cases underwent immunohistochemical staining. Four of these (aged 3, 16, 34, and 44 years) were anaplastic lymphoma kinase- (ALK-) positive.

### 3.5. Treatment and Follow-Up

Complete resection of the lesion was performed on twelve patients (12/13), and the final patient refused treatment because of the widespread infiltration of the tumor. After four recurrences, one lesion was diagnosed as low-grade malignant myofibrosarcoma. There was no metastasis after surgery in any of the seven patients during the follow-up period, which currently ranges from 17 months to 9 years. The patient who refused treatment was lost to follow-up.

## 4. Discussion

Inflammatory myofibroblastic tumor (IMT) is a rare type of myofibroblastic tumor. It was first reported as occurring in the lungs by Brunn in 1939 [7]. Because its clinical and radiological manifestations are like those of malignant tumors, it was designated an inflammatory pseudotumor by Umiker and Iversin in 1954 [8]. Due to its histopathological diversity, it has many synonyms, such as inflammatory pseudotumor, plasma cell granuloma, and inflammatory myofibroblastic hyperplasia. Over the past two decades, IMT has been classified as an intermediate neoplastic lesion (with occasional metastasis). It primarily affects children and adolescents. The clinical symptoms include fever, weight loss, pain, anemia, and thrombocytosis. One case in this group whose lesion was located on the bladder wall presented with gross hematuria and no other symptoms.

IMT occurs throughout the body, most frequently in the peritoneal cavity, retroperitoneal space, and lungs [9]. It is rarely located in the soft tissues of the extremities, bones, and joints and has only been reported in case reports [10–18]. A soft tissue extremity lesion has only been noted in one report [16]. Tumors located in bones are primarily located in the temporal bone and jaw, and there are few radiological descriptions of these lesions. This study collected thirteen cases of IMT localized in soft muscle tissue and bones, and it is a relatively large case analysis for IMT appearing in uncommon sites.

The etiology and biology of IMT are still not known in detail. The literature shows that certain cases are possibly exaggerated reactions to inflammation, trauma, and surgery, whereas some cases are associated with other malignant lesions or autoimmune diseases [19, 20]. The activation of ALK is related to the proliferation of malignant tumors. Approximately 50% of IMT presents with ALK overexpression, which is thought to be the etiology of these tumors [21, 22]. IMT shows the proliferation of mesenchymal cells after inflammatory stimuli, particularly in some elderly patients, whereas in children it manifests with chromosomal abnormalities that include ALK gene rearrangement [23]. Therefore, adult IMT may originate from an inflammatory process, and pediatric IMT may result from a tumorous process. No case in this group had a history of infection or trauma. After immunohistochemical staining, four cases presented as ALK-positive. The ages of these two cases were 34 and 44 years, demonstrating that adult IMT can produce a neoplastic response. Stimulation from infection, trauma, and other malignant tumors is not the causes of IMT in this group, and it is still unknown whether there are other forms of pathogenesis.

ALK gene rearrangement not only helps in identifying the characteristics of IMT, but also provides the opportunity to perform targeted therapy on this rare but sometimes aggressive tumor. Because of its inert biological behavior, the value of ALK-mediated target therapy for IMT treatment is limited. Although some reports showed that IMT can subside spontaneously or respond to drug therapy [19], complete surgical resection is still always proposed.

In general, IMT has a good prognosis, but its capsule is usually incomplete and surrounds important tissues and organs, and it cannot be completely resected, resulting in recurrence. Retrospective analysis indicates a recurrence rate of 31% in ALK-negative extrapulmonary IMT, and there is a 69% recurrence rate in ALK-positive IMT [21], indicating that the presence of ALK-positive extrapulmonary IMT can be an important indication of recurrence. Three cases in this group recurred after operations. Two of these were ALK-positive. The recurrence time ranged from 7 to 14 months. One case recurred eight times in eight years.

Only a tiny minority of IMT cases have the opportunity to transform into malignant tumors and give rise to distant metastases. After four recurrences, one case in this group transformed into a low-grade myofibrosarcoma, which was histologically similar to IMT. The only difference between them is the presence of inflammatory cells, the presence of cellular pleomorphism, and the possibility of local infiltration and distant metastasis. Thus far, no case in this group has developed distant metastases.

In a few reports, MRI scans have a low specificity when it comes to diagnosing IMT in soft tissue. There are three histological subtypes of IMT: (1) inflammatory type, which is characterized by a great deal of mucus and the presence of vascular and inflammatory mediators, such as granulation tissue and proliferative fasciitis; (2) cellular type, which is characterized by many compact spindle cells infiltrated with inflammatory cells, similar to fibrous histiocytoma or fibromatosis; and (3) few-cell type, which is characterized by a high amount of compact collagen, similar to desmoid tumors or scars [24].

The constituent ratio of mucus, inflammatory cells, and fibrous tissue may affect the enhancement mode and signal intensity of the tumors on MRI scanning. When mucus is abundant in the inflammatory type of IMT, MRI scans show the lesion with iso- or slight hyperintensity on T1WI compared with muscle, and bright hyperintensity on T2WI. The feature of hypointensity on both T1WI and T2WI of few-cell IMT shows that this subtype is primarily comprised of collagen fibers. The signal intensity of cellular IMT on T1WI and T2WI lies between the inflammatory type and few-cell type, but its degree of enhancement is more apparent. Hyperintensity on T2WI and more obvious enhancement after contrast-enhancement may mean that this fibroblastic lesion grows faster and becomes more aggressive than in the inflammatory type and few-cell type. Hypointensity on T2WI and no obvious enhancement after contrast-enhanced MRI suggest that fibroblastic activity is not obvious and that the lesion is more stable [25]. Eight cases in this group underwent MRI scans, and all the tumors appeared hyperintense on T2WI, whereas the ratio and distribution of cells and matrices in the tumors were histopathologically different. This suggests that MRI signaling is not precise in evaluating different kinds of histological components. But on MRI scanning, the boundary of the IMT is clear and shows peritumoral edema. The phenomenon that morphologically benign tumors show peritumoral edema may be a feature of IMT.

There is a lack of a blood supply in the center of the tumor, so that is where the area of vascular necrosis is usually located. Compared to the tumor area, the vascular necrosis shows more hyposignal intensity on T1WI, more hypersignal intensity on T2WI, and no enhancement on Enhanced MR scans. It is worth noting that tumor cells can be seen histologically in the inflammatory edema area on MRI, suggesting that this area should be included in surgical resections.

CT has good diagnostic value for the calcification of IMT lesions and the peripheral sclerotic margin of the lesions. The osteosclerosis surrounding the tumor is well-defined. The sclerotic border shows the inflammatory characteristics of uniform high density, different widths, and a blunt lateral edge of sclerotic band and the like, features which are different from the thin-layer sclerosis of benign tumors. However, no cases in this group showed any calcification, in contrast to low-grade myofibrosarcoma (LGMS), which has a higher probability of calcification, though it is difficult to identify histopathologically. All the bone IMT cases in this group showed dissolvent bone destruction, even though IMT is a pathologically intermediate and less aggressive tumor.

This group of IMT cases demonstrated complete cortex destruction and the presence of parosteal soft tissue masses on CT scans. The boundaries between the mass and normal bone are vague, which usually suggests that the tumor is more aggressive. Of note, no case in this group showed a periosteal reaction, which is similar to low level glioblastoma multiforme. The masses of two cases located in the frontal bone invaded large pieces of the endocranium and demonstrated dural enhancement on CT scan, which is similar to the dural tail sign, suggesting that IMT possesses a certain invasiveness. This also suggests that IMT was invasive to some extent. It has been reported that intratumor calcification in patients with IMT is helpful for diagnosis but this needs to be distinguished from inflammatory dead bone. No intratumor calcification was found in this group of patients. On CT of soft tissue, IMT showed a soft tissue mass, enhancement after contrast, and indistinct margins. Detection of peritumor edema needs to correlate with MRI.

## 5. Conclusion

Unlike in previous reports, the radiological manifestations of IMT located in soft tissues and bones are like those of benign tumors in shape, but the peritumoral edema, parosteal soft tissue, and invasive rim of IMT are like the features of malignant tumors. For the diagnosis of IMT, we should compare various radiological methods to achieve an accurate diagnosis. Pathological examination should be primarily based on the proliferation of myofibroblasts, and the possibility of this disease should be considered when it is accompanied by inflammatory changes. It is worth noting that tumor cells can be seen histologically in the inflammatory edema area on MRI, suggesting that the edema area should be included in surgical resections. Given that there are few cases in this study, all with various imaging protocols, a study of a larger group of patients with a uniform imaging protocol will help in deepening our understanding of this disease.

## Figures and Tables

**Table 1:** The clinical and imaging manifestations of IMT.

Number	Age	Sex	Location	Medical history	Size (cm)	Manifestation of CT	CT value (precontrast)	CT value (postcontrast)	T1WI	T2WI	Internal mass enhancement	Follow-up	ALK
1	27	F	Right maxilla	5 months	3.3 × 4.0	Low density oval mass	32–35 Hu	42–54 Hu	/	/	/		/

2	44	F	Right thigh	Recurrence 8 times in 8 years	5.6 × 7.4	/	/	/	Slight low signal	Heterogeneous high signal with dark internal septation	Heterogeneous	IMT translate into LGMS after recurrence of 3 times	Positive

3	18	M	Left shoulder	Recurrence after 2 years of operation	6.5 × 7.3	Low density lobulated mass	15–34 Hu	52–60 Hu	Slight high signal	High signal	Homogenous	Recurrence after 3 years of operation	/

4	3	F	Frontal bone	6 months	2.0 × 2.2	Isodensity round mass	67 Hu	/	/	/	/		Positive

5	54	M	Left thigh	2 months	4.1 × 6.9	/	/	/	/	/	/		/

6	47	M	Right groin	Recurrence after 14 months of operation	5.2 × 6.5	Isodensity lobulated mass	36–45 Hu	43–66 Hu	Iso signal	Iso signal	Heterogeneous	Recurrence after 29 months of operation	Negative

7	17	M	Right mandible	1 month	3.2 × 3.2	Low density oval mass	37–47 Hu	70–94 Hu	/	/	/	/	Negative

8	16	F	Left thigh	1 month	5.3 × 8.7	/	/	/	Slight high signal, with hemorrhage	High signal, with hemorrhage	Rim enhancement	/	Positive

9	34	M	Left groin	20 days	2.0 × 2.4	/	/	/	Low signal	High signal	Homogeneous	/	Positive

10	48	F	Right buttocks	3 months	1.8 × 3.0	/	/	/	Slight low signal	High signal	Homogeneous	/	/

11	48	F	Right intertrochanteric	7 months	3.0 × 4.7	Expansive damage with hyperosteogeny	/	/	/	/	/	/	/

12	59	M	Bladder	1 year	3.3 × 4.0	Low density lobulated mass	35 Hu	66 Hu	/	/	/	/	Negative

13	10	M	Frontal bone	5 months	2.1 × 2.4	Low density oval mass	26 Hu	41 Hu	Low signal	High signal		/	/
