# Corrigendum to “The Clinical and Radiological Characteristics of Inflammatory Myofibroblastic Tumor Occurring at Unusual Sites

**DOI:** 10.1155/2020/1831650

**Published:** 2020-12-12

**Authors:** Xuewei Zeng, Genggeng Qin, Huayi Huang, Jun Li, Jiayou Peng, Jiaxiong Zhang

**Affiliations:** ^1^Department of Radiology, Foshan Hospital of Traditional Chinese Medicine, Foshan 528000, China; ^2^Department of Radiology, Nanfang Hospital, Southern Medical University, China

In the article titled “The Clinical and Radiological Characteristics of Inflammatory Myofibroblastic Tumor Occurring at Unusual Sites” [1], Dr. Genggeng Qin was missing from the author list. Dr. Qin has contributed to the study by conceiving, designing, and planning the study and to provide image and should be listed as the corresponding author of this article. The corrected author list is shown above.

The authors also wish to correct the acknowledgements section as follows:

“This work was supported by the Key Specialty Construction of the Clinical Project of Foshan (Fspy3-2015019) and the Science and Technology Key Project of Foshan (2017AB002711). We appreciate the image of the case from Nanfang Hospital, Southern Medical University.”

## References

[1] Zeng X., Huang H., Li J., Peng J., Zhang J. (2018). The clinical and radiological characteristics of inflammatory myofibroblastic tumor occurring at unusual sites. *BioMed Research International*.

